# Treatment outcomes of cetuximab-containing regimen in locoregional recurrent and distant metastatic head and neck squamous cell carcinoma

**DOI:** 10.1186/s12885-022-10440-7

**Published:** 2022-12-20

**Authors:** Tien-Hua Chen, Yi-Ying Pan, Tsung-Lun Lee, Ling-Wei Wang, Shyh-Kuan Tai, Pen-Yuan Chu, Wen-Liang Lo, Cheng-Hsien Wu, Muh-Hwa Yang, Peter Mu-Hsin Chang

**Affiliations:** 1 Division of Medical Oncology, Department of Oncology, Taipei Veterans General Hospital, No.201, Sec. 2, Shipai Rd., Beitou District, Taipei, Taiwan 11217 Republic of China; 2 Department of Medicine, School of Medicine, National Yang Ming Chiao Tung University, Taipei, Taiwan; 3 Division of Radiation Oncology, Department of Oncology, Taipei Veterans General Hospital, Taipei, Taiwan; 4 Department of Otolaryngology, Taipei Veterans General Hospital, Taipei, Taiwan; 5 Department of Dentistry, School of Dentistry, National Yang-Ming University, Taipei, Taiwan; 6 Division of Oral and Maxillofacial Surgery, Department of Stomatology, Taipei Veterans General Hospital, Taipei, Taiwan; 7 Institute of Clinical Medicine, National Yang Ming Chiao Tung University, Taipei, Taiwan; 8 Institute of Biopharmaceutical Sciences, National Yang Ming Chiao Tung University, Taipei, Taiwan

**Keywords:** Cetuximab, Cetuximab-based chemotherapy, Locoregional recurrence, Distant metastasis, Recurrent/metastatic squamous cell carcinoma of head and neck

## Abstract

**Background:**

Recurrent/ metastatic squamous cell carcinoma of head and neck (R/M SCCNH) is still a difficult-to-treat disease with poor clinical outcomes and limited treatment choices. In view of locoregional recurrent versus distant metastatic SCCHN, the therapeutic efficacy of cetuximab-containing regimen and relevant prognostic factors for these two groups may be different. Thus, the aim of this study was to explore the treatment outcomes of cetuximab-containing regimen in locoregional recurrent and distant metastatic SCCHN groups, and to identify clinical factors correlated with better survival outcomes.

**Methods:**

From 2016 to 2020, patients with R/M SCCHN who received cetuximab-containing regimen in our institute were enrolled in this study. Clinical outcomes including overall survival (OS), progression-free survival (PFS), objective response rate (ORR) and disease control rate (DCR) were evaluated in both locoregional recurrence and distant metastasis groups. Exploratory analysis were conducted to investigate major clinical features associated with better outcomes.

**Results:**

A total of 107 patients with locoregional recurrent SCCHN (*N* = 66) and distant metastatic SCCNH (*N* = 41) who received cetuximab-containing regimen were enrolled in this retrospective study. Patients with oral cavity cancer and patients with disease recurrence within 6 months after radiation therapy were significantly increased in locoregional recurrence group. The median OS (15.6 vs. 9.7 months, *P* = 0.004) and PFS (5.8 months vs. 4.2 months, *P* = 0.008) were longer in locoregional recurrence group than in distant metastasis group. In multivariate analysis of clinical features, locoregional recurrence was still an important risk factor associated with better OS (Hazzard ratio (HR) 0.64, *p* = 0.06) and PFS (HR 0.67, *p* = 0.075). In addition, a trend of favorable disease control rate (DCR; 62.5% vs. 45.0%, *p* = 0.056) was noted in locoregional recurrence group. In locoregional recurrence group, prior salvage surgery was associated with longer OS (HR = 0.24, *P* = 0.008) and PFS (HR = 0.30, *P* = 0.005).

**Conclusion:**

SCCHN with locoregional recurrence is associated with better disease control and survival outcomes comparing to distant metastatic SCCHN when treated with cetuximab-containing regimen. Salvage surgery for locoregional recurrence may further improves clinical outcome.

**Supplementary Information:**

The online version contains supplementary material available at 10.1186/s12885-022-10440-7.

## Key points

Questions: Is there difference in outcomes of cetuximab-containing regimen between locoregional recurrence and distant metastatic squamous cell carcinoma of head-and-neck (SCCHN)?

Findings: In this retrospective study, SCCHN patients who received cetuximab-containing regimen had significantly better median overall survival (15.6 vs. 9.7 months, *P* = 0.004) and median progression-free survival (5.8 months vs. 4.2 months, *P* = 0.008) in locoregional recurrence group than in distant metastasis group.

Meaning: Cetuximab-containing regimen is the treatment of choice in locoregional recurrent SCCHN.

## Introduction

Squamous cell carcinoma of head and neck (SCCHN) is one of the most common cancers worldwide, with more than 900,000 new cases and over 400,000 deaths annually [1]. The prevalence of SCCHN is high in Asian countries, especially in Taiwan. Based on the statistics from the National Cancer Registry in Taiwan, the incidence of SCCHN continues to rise, ranking fourth and seventh most common malignancy in men and both sexes, respectively [2]. Despite an intensive combination of surgery, radiation therapy, and platinum-based chemotherapy for curative treatment, the recurrence rate is 20–30% in early stage SCCHN and up to 50% in locally advanced SCCHN [3–7]..

Currently, the treatment options of R/M SCCHN is still limited, and the survival outcomes are still poor [8, 9]. Combining cetuximab with systemic chemotherapy had significantly improved treatment outcomes in two randomized clinical trials [10, 11]. In EXTREME study, by incorporating cetuximab with platinum and fluorouracil, the risk of death significantly decreased by 20%, the risk of disease progression decreased by 36%, and the response rate increased 16%. In the TPExtreme study, cetuximab combined with docetaxel and cisplatin resulted in similar overall survival and progression free survival outcomes with EXTREME regimen, and with an objective response rate of 57%. Hence, cetuximab combined with chemotherapy is still one of the treatment of choice for R/M SCCHN. However, despite the effort in researching optimal combination of cetuximab and chemotherapy, the median survival of R/M SCCHN treated with cetuximab-based chemotherapy was only around one year, and the long term survival is still dismal. Hence, it is of utmost importance to evaluate prognostic factors for cetuximab treatment. In two prior large and detailed retrospective studies, multiple prognostic factors were identified, and among them distant metastasis was identified as a strong factor for poor survival in HNSCC [12, 13]. However, whether distant metastasis maintained to be major prognostic factors for R/M SCCHN treated with cetuximab-containing regimen remains to be elucidated.

Based on this rationale, the aim of our study was to explore the difference of cetuximab-containing regimen efficacy between locoregional recurrence and distant metastasis for R/M SCCHN in our institute. Potential prognostic factors were also evaluated in this study.

## Methods

### Study design and participants

This retrospective study was conducted in accordance with the Declaration of Helsinki and approved by the Institutional Review Board of Taipei Veterans General Hospital (IRB: #2020–08-013 BC). From 2016 to 2020, patients who were diagnosed of R/M SCCHN and had received cetuximab-containing regimen were enrolled in this study. The inclusion criteria were as follows: (1) Patient aged older than 18 years who had recurrence after primary CCRT or adjuvant CCRT, disease refractory to induction chemotherapy, or distant metastasis at initial diagnosis, (2) The recurrence of SCCHN was histologically confirmed, (3) Patient had at least one measurable lesion identified by computed tomography (CT) or magnetic resonance imaging (MRI), (4) Patient had received cetuximab-containing regimen after diagnosis of R/M SCCHN. Locoregional recurrence group was defined as the recurrence at the same site as the original (primary) tumor, and without second primary malignancy or distant metastasis. Distant metastasis group was defined as the involvement of distant organs, with or without locoregional recurrence. Patients with unresectable disease or comorbidities are regarded as ineligible for salvage surgery. Salvage surgery included two types of procedures. One was primary section with or without neck dissection, and the other was neck dissection alone. Reirradiation (re-RT) was defined as adjuvant treatment to salvage surgery, or salvage treatment to unresectable disease.

### Primary and secondary endpoints

The primary endpoint of this study was to assess overall survival (OS) in R/M SCCHN patients treated with cetuximab-containing regimen. The secondary endpoints included the progression-free survival (PFS), objective response rate (ORR) and disease control rate (DCR). Treatment response to cetuximab-containing regimen was assessed by the revised Response Evaluation Criteria in Solid Tumors (RECIST) version 1.1 [14] OS was defined as the interval between the date of R/M SCCHN first receiving cetuximab-containing regimen and the date of death or the last follow up before censoring. PFS was defined as the interval between the date of first administration of cetuximab-containing regimen and the date of disease progression, or death from any cause. The abovementioned treatment efficacy was evaluated in overall cohort, in locoregional recurrence group, and in distant metastasis group, respectively.

### Statistical analyses

Continuous data were presented as median with inter-quartile range (IQR), and categorical data were presented as the number and percentage (%). Pearson’s chi-square test was used to examine the difference between the locoregional recurrence group and distant metastasis group in age, sex, smoking, betel nuts, previous systemic treatment, treatment after recurrence (cetuximab-containing regimen, salvage surgery, re-RT). Fisher’s exact test was used to analyze the difference of primary tumor location between the two groups. Cox proportional hazards regression analysis was performed to identify potential prognostic factors affecting OS and PFS in the whole R/M SCCHN cohort and respectively in the locoregional recurrence group and distant metastasis group. Prognostic factors with *p*-value< 0.10 in univariate analysis will be further evaluated by multivariate analysis. OS and PFS curves were estimated by Kaplan-Meier survival analysis, and the differences between groups were determined by log-rank test. All statistical analyses were performed using SPSS version 22.0.

## Results

### Demographics and clinical characteristics of the patients

Patient characteristics are summarized in Table 1. A total of 107 patents with R/M SCCHN were treated with cetuximab-containing regimen, with a median age of 60.2 years (range 58.5–62.1 years). Most patients were men (93.5%) and have a history of cigarette smoking (60.7%) and betel nuts chewing (83.2%), and good performance status (ECOG = 0, 66.3%). The primary tumor was mainly located in the oral cavity (50.5%), followed by hypopharynx (20.6%), oropharynx (17.8%), larynx (8.4%), and others (2.8%). Differentiation of tumor was documented in 77 patients, and mostly well to moderately differentiated (71.9%). Previous systemic treatment included neoadjuvant chemotherapy (37.4%), primary CCRT (43.9%) and adjuvant CCRT (46.7%). During the treatment course of cetuximab-containing regimen, 63.6% patients received EXTREME regimen (cetuximab combined cisplatin/carboplatin and infusional 5-fluorouracil). Nearly half of all cases had recurrence within 6 months after RT (46.7%), and the other half occurred more than 6 months after RT (45.8%). R/M SCCHN patients were divided into subgroups according to the location of disease recurrence/metastasis: recurrence in the locoregional area only (locoregional recurrence group, *n* = 66) or distant metastasis with or without locoregional recurrence (distant metastasis group, *n* = 41). There was no statistical difference between the two groups in age, sex, history of smoking and betel nuts chewing, performance status, differentiation of tumor, previous systemic treatments, and whether received EXTEME regimen or not. Increased proportion of oral cavity SCCHN and proportion of recurrence within 6 months after radiation therapy were noted in locoregional recurrence group. Salvage surgery and re-RT were only performed in 21.2 and 22.7% of patients within locoregional recurrence group, respectively. Among the patients who received re-RT, re-RT was adjuvant treatment to salvage surgery in 6 cases, and was salvage treatment to non-resectable disease in 9 cases.

**Table 1:** Demographics and clinical characteristics of the study cohort (*N* = 107)

	Total	Locoregional recurrence	Distant metastasis	*p*-value
	(*n* = 107)	(*n* = 66)	(*n* = 41)	
Age, years (IQR)	60.2 (58.5–62.1)	60.5 (58.2–62.9)	59.9 (57.0–62.7)	0.710
Sex				0.289
Male	100 (93.5%)	63 (95.5%)	37 (90.2%)	
Female	7 (6.5%)	3 (5.0%)	4 (9.8%)	
Smoking				0.712
Former or current	65 (60.7%)	41 (62.1%)	24 (58.5%)	
Never	42 (39.3%)	25 (37.9%)	17 (41.5%)	
Betel nuts				0.264
Former or current	89 (83.2%)	57 (86.4%)	32 (78.0%)	
Never	18 (16.8%)	9 (13.6%)	9 (22.0%)	
Performance status				0.110
ECOG = 0	71 (66.3%)	40 (60.6%)	31 (75.6%)	
ECOG≧1	36 (33.6%)	26 (39.4%)	10 (24.4%)	
Primary tumor location				**0.043**^¢^
Oral cavity	54 (50.5%)	40 (60.6%)	14 (34.1%)	
Oropharynx	19 (17.8%)	7 (10.6%)	12 (29.3%)	
Hypopharynx	22 (20.6%)	11 (16.7%)	11 (26.8%)	
Larynx	9 (8.4%)	6 (9.1%)	3 (7.3%)	
Other sites†	3 (2.8%)	2 (3.0%)	1 (2.4%)	
Differentiation of tumor				0.248
Well to moderate	68 (63.5%)	38 (57.6%)	30 (73.2%)	
Poor	9 (8.4%)	6 (9.1%)	3 (7.3%)	
Not available	30 (28.0%)	22 (33.3%)	8 (19.5%)	
Previous systemic treatment^s^				0.086
Neoadjuvant chemotherapy	40 (37.4%)	27 (31.8%)	13 (25.0%)	
Primary CCRT	47 (43.9%)	33 (38.8%)	14 (26.9%)	
Adjuvant CCRT	50 (46.7%)	25 (29.4%)	25 (48.1%)	
Treatment after recurrence				0.663
EXTREME regimen^γ^	68 (63.6%)	43 (65.2%)	25 (61.0%)	
Salvage surgery	14 (21.2%)	14 (21.2%)	–	
Reirradiation	15 (22.7%)	15 (22.7%)	–	
Recurrence after RT				**0.035**
< 6 months	50 (46.7%)	37 (56.1%)	13 (31.7%)	
≧ 6 months	49 (45.8%)	26 (39.4%)	23 (56.1%)	
Not available	8 (7.5%)	3 (4.5%)	5 (12.2%)	

Abbreviations: *IQR* interquartile range, *R/M SCCHN* recurrent or metastatic squamous cell carcinoma of the head and neck, *CCRT* concurrent chemoradiotherapy

Data are presented with median (IQR) or *n* (%). Bold indicates statistically significant at *p* < 0.05

†External auditory canal in one patients and neck lymph node in two patients

¶ Patient may have received systemic treatment in more than one context

¢ Fisher’s exact test

γFor those who did not receive EXTREME regimen, the combination of cetuximab-containing regimen included: MEMOCLUB (*n* = 28, a frequently used regimen in platinum-refractory HNSCC adapted to local practice [15], methotrexate (*n* = 3), cetuximab alone (*n* = 5), pembrolizumab (*n* = 1), pembrolizumab + paclitaxel (*n* = 1), and TPF (*n* = 1)

### Treatment outcomes of cetuximab-based chemotherapy in patients with R/M SCCHN

**Fig. 1:**
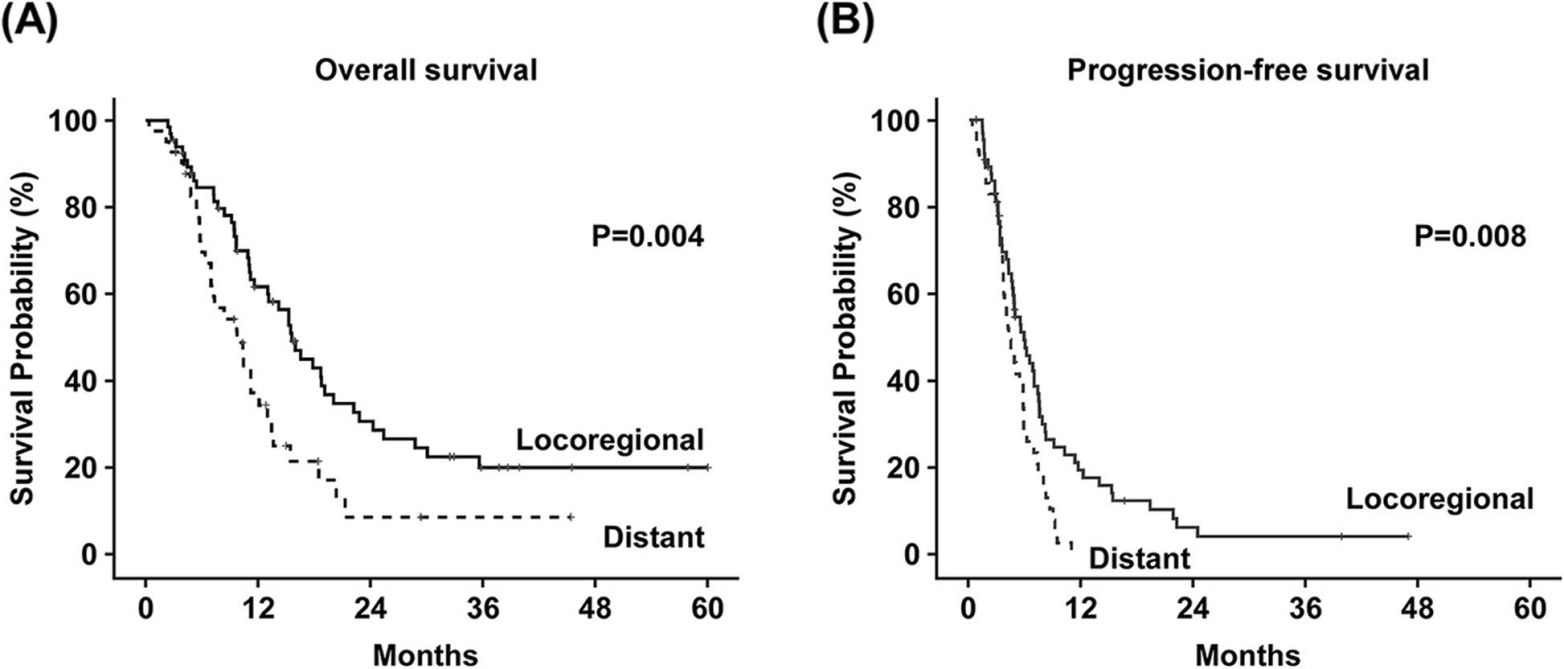
Kaplan-Meier survival plot for patients with locoregional recurrence and distant metastasis. **A** Overall survival. **B** Progression-free survival. Compared with the distant metastasis group, cetuximab-based chemotherapy showed significantly better OS and PFS in the local recurrence group

For R/M SCCHN patients who had received cetuximab-containing regimen, the median OS of overall cohort was 13.0 months (95% confidence interval (95%CI), 10.3–15.8 months), and the median PFS of overall cohort was 5.0 months (95% CI 4.0–5.9 months). The median OS (15.6 vs. 9.7 months, *P* = 0.004) and median PFS (5.8 months vs. 4.2 months, *P* = 0.008) were significantly longer in locoregional recurrence group than in distant metastasis group. (Fig. 1) In the distant metastasis group, 15 patients had distant metastasis only, and 26 patients had concurrent locoregional recurrence/ distant metastasis. The detailed median OS and PFS of distant metastasis only group and of concurrent locoregional recurrence/ distant metastasis group were shown in supplementary Fig. 1 and supplementary Table 1.

Tumor responses to cetuximab-containing regimen are shown in Table 2. The best ORR in overall population was 33.7%, with 1 patient achieving complete response (CR) and 34 patients achieving partial response (PR). The best DCR was 56.7%, which included further 34 patients with stable disease (SD). In locoregional recurrence group, the best ORR and DCR were 35.9 and 62.5%, respectively. In distant metastasis group, the best ORR and DCR were 30.0 and 45.0%, respectively. Compared with distant metastasis, locoregional recurrence was associated with higher DCR to cetuximab-based chemotherapy (62.5% vs. 45.0%), although it did not reach statistical significance (*P* = 0.056).

**Table 2:** Treatment response to cetuximab-based chemotherapy

	Total	Locoregional recurrence	Distant metastasis	*p*-value
	(*n* = 104)	(*n* = 61)	(*n* = 40)	
**Treatment Response**				0.169
CR	1 (1.0%)	1 (1.6%)	0 (0.0%)	
PR	34 (32.7%)	22 (34.4%)	12 (30.0%)	
SD	24 (23.1%)	18 (28.1%)	6 (15.0%)	
PD	45 (43.3%)	23 (35.9%)	22 (55.0%)	
ORR	35 (33.7%)	23 (35.9%)	12 (30.0%)	0.533
DCR	59 (56.7%)	41 (62.5%)	18 (45.0%)	0.056

Abbreviations: *CR* complete response, *PR* partial response *SD* stable disease, *PD* progressive disease, *ORR* objective response rate, *DCR* disease control rate. The tumor response of 3 patients could not be assessed. Data are presented with median (IQR) or *n* (%). Bold indicates statistically significant at *p* < 0.05

### Independent prognostic factors for OS and PFS in SCCHN patients with locoregional recurrence or distant metastasis

Cox regression analyses of clinical and treatment characteristics were performed to determine independent prognostic factors for OS and PFS (Table 3). Univariate analysis showed that pattern of disease recurrence (locoregional recurrence only or distant metastasis) and salvage surgery were the prognostic factors for both OS and PFS. In the multivariate analysis, locoregional recurrence still showed trend of decreased risk of death (HR = 0.64, *p* = 0.06) and of disease progression (HR = 0.67, *p* = 0.075), although without statistical significance. Furthermore, for locoregional recurrence without salvage surgery group, the OS was significantly longer compared to distant metastasis group (15.3 vs. 9.7 months, *p* = 0.048; supplementary Fig. 2). Salvage surgery remained to be a significant prognostic factor in multivariate analysis. Subgroup analyses were further performed to examine the factors affecting OS and PFS in the locoregional recurrence group and the distant metastasis group, respectively. Multivariate analyses showed that salvage surgery in the locoregional recurrence group was significantly associated with improved OS (HR = 0.24, *P* = 0.006, Table 4) and PFS (HR = 0.30, *P* = 0.005, Table 5). Additionally, male sex was associated with significantly improved PFS in the locoregional recurrence group (HR = 0.13, *P* = 0.003, Table 5). For the distant metastasis group, recurrence < 6 months after RT was identified to significantly increased risk of death and disease progression.

**Table 3:** Cox regression analysis of risk factors for OS and PFS

Variables	OS				PFS			
	Univariate analysis		Multivariate analysis		Univariate analysis		Multivariate analysis	
	HR (95% CI)	*p*-value	HR (95% CI)	*p-*value	HR (95% CI)	*p*-value	HR (95% CI)	*p*-value
Age (> 60 vs. ≤60)	0.99 (0.63–1.56)	0.533			1.16 (0.77–1.74)	0.476		
Sex (Male vs. Female)	0.64 (0.28–1.48)	0.935			0.53 (0.24–1.16)	0.111		
Smoking (Former/current vs. Never)	0.83 (0.52–1.32)	0.425			0.84 (0.48–1.46)	0.539		
Betel nuts (Former/current vs. Never)	1.21 (0.64–2.28)	0.567			0.85 (0.56–1.29)	0.444		
Performance status scale (ECOG ≧1 vs. 0)	1.38 (0.87–2.20)	0.176			1.20 (0.78–1.83)	0.414		
Primary tumor location (Oral cavity vs. others)	0.77 (0.49–1.21)	0.249			0.80 (0.53–1.21)	0.299		
Primary tumor location (Hypolarynx/ larynx vs. others)	1.31 (0.80–2.13)	0.279			1.39 (0.88–2.19)	0.156		
Previous systemic treatment								
Neoadjuvant chemotherapy (Yes vs. No)	1.18 (0.74–1.88)	0.500			0.94 (0.61–1.43)	0.756		
Primary CCRT (Yes vs. No)	0.93 (0.59–1.47)	0.761			0.83 (0.55–1.25)	0.826		
Adjuvant CCRT (Yes vs. No)	0.88 (0.56–1.39)	0.593			1.07 (0.71–1.60)	0.750		
Disease recurrence (Locoregional vs. Distant metastasis)	0.51 (0.32–0.82)	**0.005**	0.64 (0.40–1.02)	0.060	0.56 (0.37–0.87)	**0.009**	0.67 (0.43–1.04)	0.075
Salvage surgery (Yes vs. No)	0.21 (0.08–0.58)	**0.003**	0.24 (0.09–0.69)	**0.007**	0.30 (0.14–0.70)	**0.003**	0.34 (0.15–0.77)	**0.010**
Recurrence after RT (< 6 months vs. > 6 months)	1.44 (0.91–2.30)	0.122			1.35 (0.89–2.06)	0.162		

Abbreviations: *OS* overall survival, *PFS* progression-free survival, *HR* hazard ratio, *CI* confidence intervals, *ECOG* Eastern Cooperative Oncology Group, *CCRT* concurrent chemoradiotherapy, *RT* radiation therapy. Bold indicates statistically significant at *p* < 0.05

**Table 4:** Univariate and multivariate analyses of risk factors on OS for locoregional recurrence and distant metastasis

Variables	Locoregional recurrence				Distant metastasis			
	Univariate		Multivariate		Univariate		Multivariate	
	HR (95% CI)	*p*-value	HR (95% CI)	*p*-value	HR (95% CI)	*p*-value	HR (95% CI)	*p*-value
Age (> 60 vs. ≤60)	1.04 (0.58–1.87)	0.897			0.87 (0.43–1.74)	0.685		
Sex (Male vs. Female)	0.35 (0.11–1.13)	0.078	0.56 (0.16–1.92)	0.354	1.24 (0.37–4.12)	0.731		
Smoking (Former/current vs. Never)	1.17 (0.46–2.96)	0.742			1.71 (0.69–4.24)	0.247		
Betel nuts (Former/current vs. Never)	0.71 (0.39–1.29)	0.254			1.04 (0.50–2.17)	0.909		
Performance status scale (ECOG ≧1 vs. 0)	1.82 (1.01–3.30)	**0.048**	1.54 (0.83–2.86)	0.175	1.17 (0.50–2.73)	0.715		
Primary tumor location (Oral cavity vs. Others)	0.69 (0.38–1.25)	0.220			1.26 (0.61–2.58)	0.537		
Primary tumor location (Hypolarynx/ larynx vs. others)	1.69 (0.87–3.28)	0.119			0.86 (0.41–1.78)	0.675		
Previous systemic treatment								
Neoadjuvant chemotherapy (Yes vs. No)	1.36 (0.75–2.48)	0.310			1.31 (0.58–2.96)	0.509		
Primary CCRT (Yes vs. No)	1.01 (0.56–1.82)	0.965			1.14 (0.53–2.46)	0.739		
Adjuvant CCRT (Yes vs. No)	0.68 (0.36–1.27)	0.224			0.85 (0.41–1.78)	0.665		
Recurrence after RT (< 6 months vs. > 6 months)	1.28 (0.70–2.33)	0.426			5.32 (2.12–13.40)	**< 0.001**		
Treatment								
Salvage surgery (Yes vs. No)	0.23 (0.08–0.66)	**0.006**	0.25 (0.10–0.72)	**0.010**	–			
Salvage radiotherapy (Yes vs. No)	0.71 (0.32–1.60)	0.408			–			

Abbreviations: *HR* hazard ratio, *CI* confidence intervals, *ECOG* Eastern Cooperative Oncology Group, *CCRT* concurrent chemoradiotherapy. Bold indicates statistically significant at *p* < 0.05

**Table 5:** Univariate and multivariate analyses of risk factors on PFS for locoregional recurrence and distant metastasis

Variables	Locoregional recurrence				Distant metastasis			
	Univariate		Multivariate		Univariate		Multivariate	
	HR (95% CI)	*p*-value	HR (95% CI)	*p*-value	HR (95% CI)	*p*-value	HR (95% CI)	*p*-value
Age (> 60 vs. ≤60)	1.25 (0.74–2.13)	0.408			0.88 (0.46–1.68)	0.695		
Sex (Male vs. Female)	0.16 (0.04–0.57)	**0.005**	0.13 (0.04–0.50)	**0.003**	1.26 (0.44–3.36)	0.670		
Smoking (Former/current vs. Never)	0.86 (0.36–2.04)	0.735			1.09 (0.51–2.32)	0.819		
Betel nuts (Former/current vs. Never)	0.85 (0.49–1.47)	0.562			0.88 (0.46–1.70)	0.709		
Performance status scale (ECOG ≧1 vs. 0)	1.56 (0.91–2.66)	0.105			0.92 (0.42–2.02)	0.827		
Primary tumor location (Oral cavity vs. Others)	0.87 (0.51–1.50)	0.615			1.13 (0.57–2.23)	0.732		
Primary tumor location (Hypolarynx/ larynx vs. others)	1.36 (0.73–2.53)	0.330			1.34 (0.68–2.62)	0.399		
Previous systemic treatment								
Neoadjuvant chemotherapy (Yes vs. No)	1.03 (0.60–1.75)	0.917			1.05 (0.49–2.23)	0.906		
Primary CCRT (Yes vs. No)	0.85 (0.50–1.45)	0.554			1.00 (0.51–1.96)	0.994		
Adjuvant CCRT (Yes vs. No)	0.88 (0.51–1.52)	0.638			1.09 (0.57–2.10)	0.798		
Recurrence after RT (< 6 months vs. > 6 months)	1.30 (0.76–2.22)	0.347			3.14 (1.43–6.89)	**0.004**		
Treatment								
Salvage surgery (Yes vs. No)	0.36 (0.16–0.81)	**0.014**	0.30 (0.13–0.69)	**0.005**				
Salvage radiotherapy (Yes vs. No)	1.07 (0.54–2.12)	0.849						

Abbreviations: *HR* hazard ratio, *CI* confidence intervals, *ECOG* Eastern Cooperative Oncology Group, *CCRT* concurrent chemoradiotherapy. Bold indicates statistically significant at *p* < 0.05

**Fig. 2:**
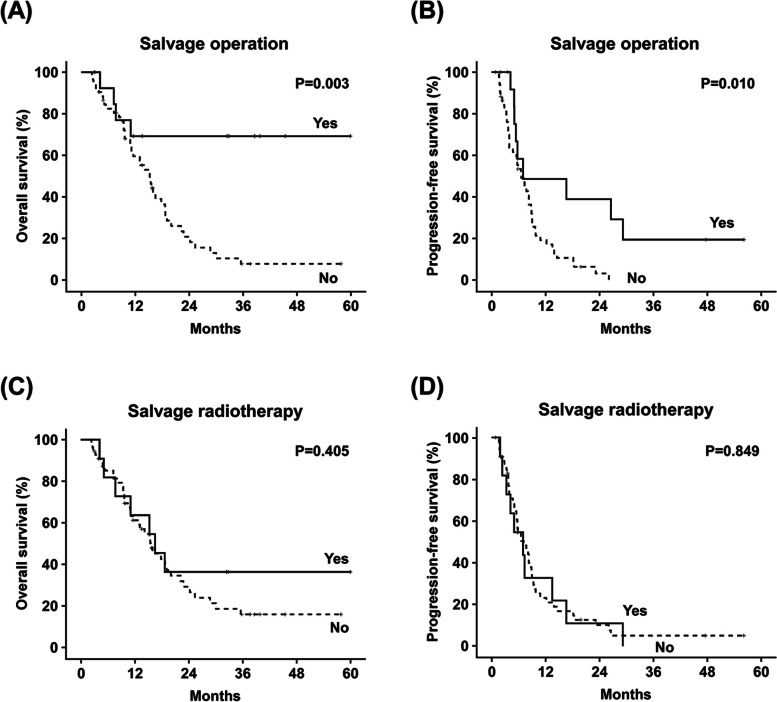
Kaplan-Meier survival analyses with log-rank test for OS and PFS of SCCHN patients with locoregional recurrence

The Kaplan-Meier curves of OS and PFS in R/M SCCHN patients who received/not received salvage surgery and re-RT were shown in Fig. 2. For R/M SCCHN patients with locoregional recurrence, salvage surgery was associated with significantly prolonged OS (median: not reached vs. 15.2 months, *P* = 0.003; Fig. 2A) and PFS (median: 5.8 months vs. 5.5 months, *P* = 0.010; Fig. 2B). For patients with or without salvage surgery, the estimated 2-year OS rate were 71.4 and 26.3%, and the estimated 5-year OS rate were 71.4 and 9.8%, respectively. The estimated 2-year PFS rate were 34.3 and 3.2%, and the estimated 5-year-PFS rate were 22.9 and 0.0%, respectively. For patients with or without re-RT, there were no significant survival difference observed in terms of OS (median: 16.5 months vs. 15.6 months, *P* = 0.405; Fig. 2C) and PFS (median: 5.8 months vs. 5.9 months, *P* = 0.849; Fig. 2D). The estimated 2-year OS rate were 36.3% vs. 35.1%, and the estimated 5-year OS rate were 36.3 and 0%, respectively. The estimated 2-year PFS rate were 13.6 and 9.1%, and the estimated 5-year-PFS rate were 0.0 and 9.1%, respectively.

## Discussion

In this study, the efficacy of cetuximab-containing regimen was evaluated in the overall R/M SCCHN cohort, in the locoregional recurrence only group, and in the distant metastasis group. Herein, a significantly longer OS and PFS were noted in the locoregional recurrence group comparing to distant metastasis group. In further univariate and multivariate analysis of clinical and treatment features, the recurrence pattern (locoregional recurrence only or with distant metastasis) proved to be an important factor affecting survival outcomes of cetuximab-containing regimen in R/M SCCHN. A trend of better DCR was also noted in locoregional recurrence group treated with cetuximab-containing regimen. Moreover, through univariate and multivariate analysis of clinical and treatment features respectively in locoregional recurrence and distant metastasis group, a favorable survival outcome was noted for those who received salvage surgery comparing to those who received re-RT. These findings support cetuximab-containing regimen as the treatment of choice in locoregional recurrent SCCHN.

In our study cohort, the median OS was 13.0 months and median PFS was 5.0 months. These survival outcomes were similar to the outcomes of cetuximab-treated arm in the EXTREME study (median OS: 10.1 months, median PFS 5.6 months) and in the KEYNOTE-048 study (median OS: 10.7 months, median PFS 5.1 months) [10, 16]. The survival outcome of the overall cohort in this study was also similar to the findings in the single arm observational ENCORE study (median OS: 10.2 months, median PFS 6.5 months) and another randomized, open-label, phase III CHANGE-2 study (median OS: 11.1 months, median PFS: 5.5 months) [17, 18]. In both KEYNOTE-048 and ENCORE study, the treatment efficacy of cetuximab-based chemotherapy in locoregional and distant metastasis group was not disclosed. In EXTREME study, the impact on OS of the cetuximab containing arm was more prominent in locoregional recurrence group (OS: HR 0.65, 95% CI 0.49–0.87) than in distant metastasis group (OS: HR 0.99, 95% CI 0.72–1.38) [19]. In CHANGE-2 study, cetuximab containing treatment also showed similar OS benefits in locoregional recurrence group (OS: HR 0.6, 95% CI 0.4–0.9) but not in distant metastasis group (OS: HR 0.7, 95% CI 0.3–1.7) or locoregional recurrence plus distant metastasis group (OS: HR 0.9, 0.5–1.8) [20]. In the prospective observational study JROSG 12–2, a trend of increased risk of death was noted in patients with lung and bone metastasis (Lung: HR 2.12, *p* = 0.12; bone: HR 2.29, *p* = 0.11) [21]. These findings are compatible with our study, which showed decreased risk of death (HR 0.51, *p* = 0.005) and disease progression (HR 0.56, *p* = 0.009) in locoregional group comparing to distant metastasis group when treated with cetuximab-containing regimen. We had further evaluated the survival outcomes separately in locoregional recurrence group, distant metastasis only group, and distant metastasis plus locoregional recurrence group. Superior OS (median OS: 15.6, 7.2, 10.4 months, *p* = 0.014) and PFS (median PFS: 5.8, 3.7, 4.4 months, *p* = 0.014) were still noted in the locoregional recurrence group (Supplementary Fig. 1).

Salvage surgery and re-RT are treatment of choice for locoregional recurrent SCCHN. One retrospective study had shown favorable outcome for locoregional recurrent SCCHN receiving salvage surgery, with estimated 5-year-OS of 42% and 5-year-PFS of 47% [22]. On the other hand, in one phase II study for locoregional recurrent or second primary SCCHN in previous RT field who received re-RT in combination with cisplatin and paclitaxel, the survival outcome was dismal with estimated 2-year OS of 25.9% [23]. In another phase III study, for locoregional recurrent SCCHN who received re-RT in combination with 5-fluorouracil and hydroxyurea, the estimated 2-year OS and 5-year-OS were only 15.2 and 3.8%, respectively [24]. Retrospective study have shown that in locoregional recurrent SCCHN, salvage surgery was associated with decreased risk of death (HR 0.37, *p* = 0.001), while no significant difference was observed when treated with re-RT [25]. Retrospective study of salvage treatment for locoregional recurrent SCCHN also disclosed a favorable outcome for those who received salvage surgery (estimated 5-year OS 48.7%) comparing to who received re-RT or chemotherapy alone (estimated 5-year OS 31.6 and 3.7%, respectively) [26]. Another retrospective study also showed similar result, with estimated 5-year-OS of 26, 0, and 0% in salvage surgery, re-RT, and chemotherapy alone group [27]. However, this difference may need to be explained with caution, since those who did not receive salvage surgery for locoregional recurrence usually had more advanced disease or poorer performance status than those who were eligible to surgery. In our study, favorable outcome for cetuximab-containing regimen was noted in those who received salvage surgery but not in those who received re-RT. These findings were compatible with previous researches. Further analyses also showed a persistent favorable OS in locoregional recurrent SCCHN patients who did not receive salvage surgery comparing to distant metastasis group (Supplementary Fig. 2, *P* = 0.048).

This study has several limitations due to the retrospective and uncontrolled nature. First, this is single-center, observational study, which may limit generalizability to other populations with different demographics or populations. Another potential limitation was that different drugs combined with cetuximab were not used as prognostic variables for further statistical analysis due to the complexity of the regimens. Future multi-center randomized controlled clinical trials with standard protocols are required to overcome the limitations of this study.

## Conclusion

SCCHN with locoregional recurrence is associated with better disease control and survival outcomes comparing to distant metastatic SCCHN when treated with cetuximab-containing regimen. Salvage surgery for locoregional recurrence may further improves clinical outcome.

## Supplementary Information

**Additional file 1: Supplementary Fig 1.** Kaplan-Meier survival curves of subgroup analysis of R/M SCCHN patients.

**Additional file 2:** **Supplementary Table 1.** OS and PFS in locoregional recurrence only, distant metastasis only, and in concurrent locoregional/ distant metastasis group.

**Additional file 3: Supplementary Fig 2.** Kaplan-Meier curves of analysis of locoregional recurrence without salvage surgery and distant metastasis only.

## Data Availability

The data used and analyzed in this study are available from the corresponding author upon reasonable request.
