# Supplementary figures and images for: Treatment outcomes of cetuximab-containing regimen in locoregional recurrent and distant metastatic head and neck squamous cell carcinoma
Source: BMC Cancer. 2022 Dec 20;22:1336. doi: 10.1186/s12885-022-10440-7 (PMC9769042; doi:10.1186/s12885-022-10440-7)

**
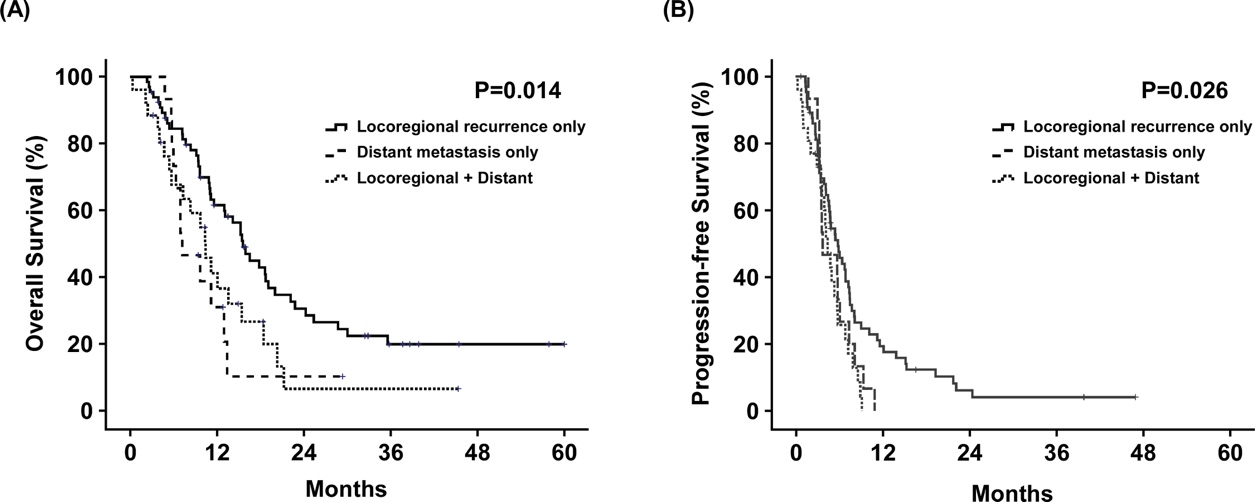
**

**Supplementary Figure 1. Kaplan-Meier survival curves of subgroup analysis of R/M SCCHN patients.**

Supplement: Supplementary file 1 — Additional file 1: Supplementary Fig 1. Kaplan-Meier survival curves of subgroup analysis of R/M SCCHN patients. [file 12885_2022_10440_MOESM1_ESM.docx]
