# Supplementary material for: Treatment outcomes of cetuximab-containing regimen in locoregional recurrent and distant metastatic head and neck squamous cell carcinoma
Source: BMC Cancer. 2022 Dec 20;22:1336. doi: 10.1186/s12885-022-10440-7 (PMC9769042; doi:10.1186/s12885-022-10440-7)
Supplement: Supplementary file 2 — Additional file 2: Supplementary Table 1. OS and PFS in locoregional recurrence only, distant metastasis only, and in concurrent locoregional/ distant metastasis group. [file 12885_2022_10440_MOESM2_ESM.docx]

**Supplementary Table 1. OS and PFS in locoregional recurrence only, distant metastasis only, and in concurrent locoregional/ distant metastasis group**

|  | **OS**  **(months, 95% CI)** | **PFS**  **(months, 95% CI)** |
| --- | --- | --- |
| Locoregional recurrence only (n=66) | 15.6 (12.2-19.1) | 5.8 (4.2-7.5) |
| Distant metastasis only (n=15) | 7.2 (4.2-10.3) | 3.7 (1.0-6.5) |
| Concurrent locoregional/ distant metastasis (n=26) | 10.4 (8.3-12.6) | 4.4 (3.3-5.5) |
| **p-value** | 0.014 | 0.026 |
