# Supplementary material for: Treatment outcomes of cetuximab-containing regimen in locoregional recurrent and distant metastatic head and neck squamous cell carcinoma
Source: BMC Cancer. 2022 Dec 20;22:1336. doi: 10.1186/s12885-022-10440-7 (PMC9769042; doi:10.1186/s12885-022-10440-7)
Supplement: Supplementary file 3 — Additional file 3: Supplementary Fig 2. Kaplan-Meier curves of analysis of locoregional recurrence without salvage surgery and distant metastasis only. [file 12885_2022_10440_MOESM3_ESM.docx]

**
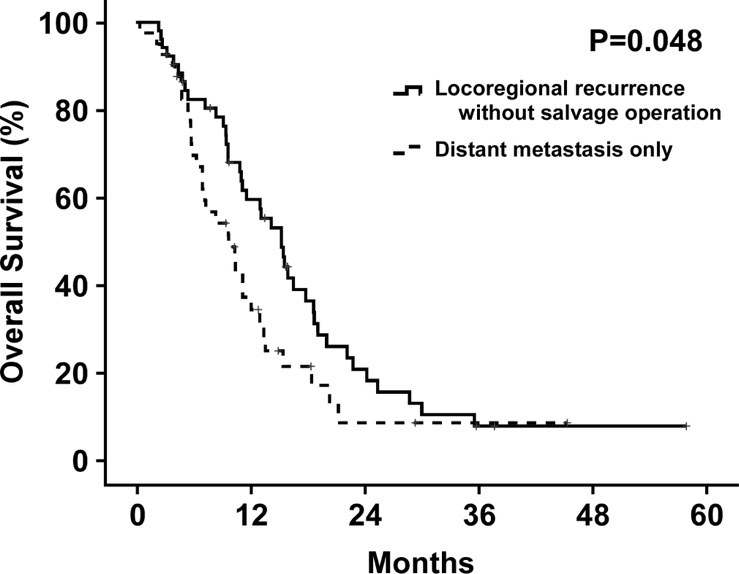
**

**Supplementary Figure 2. Kaplan-Meier curves of analysis of locoregional recurrence without salvage surgery and distant metastasis only.**
